# Catheter-Related Bloodstream Infections and Catheter Colonization among Haemodialysis Patients: Prevalence, Risk Factors, and Outcomes

**DOI:** 10.1155/2021/5562690

**Published:** 2021-06-19

**Authors:** Shamira Shahar, Ruslinda Mustafar, Lydia Kamaruzaman, Petrick Periyasamy, Kiew Bing Pau, Ramliza Ramli

**Affiliations:** ^1^Department of Medicine, Universiti Kebangsaan Malaysia Medical Centre (UKMMC), Jalan Yaacob Latif, Bandar Tun Razak, Cheras, Kuala Lumpur 56000, Malaysia; ^2^Department of Pharmacy, Universiti Kebangsaan Malaysia Medical Centre, Jalan Yaacob Latif, Bandar Tun Razak, Cheras, Kuala Lumpur 56000, Malaysia; ^3^Department of Medical Microbiology and Immunology, Universiti Kebangsaan Malaysia Medical Center, Jalan Yaacob Latif, Bandar Tun Razak, Cheras, Kuala Lumpur 56000, Malaysia

## Abstract

**Introduction:**

Catheter-related bloodstream infection (CRBSI) and catheter colonization (CC) are two complications among haemodialysis (HD) patients that lead to increased morbidity and mortality. This study aims to evaluate the prevalence of CRBSI and CC among HD patients registered at Universiti Kebangsaan Malaysia Medical Centre and to identify the factors involved by focusing on the demographic profile of the patients as well as their clinical characteristics and outcomes.

**Method:**

This is a retrospective study of end-stage renal disease patients with suspected CRBSI during the period from 1 January 2016 to 31 December 2018. Data on patients who fulfilled the blood culture criteria for CRBSI and CC diagnosis were further analysed for clinical manifestations, comorbidities, history of dialysis, catheter characteristics, and microbiological culture results. The outcomes of CRBSI and CC were also assessed. *Findings*. In the 3-year period under study, there were 496 suspected CRBSI cases with a total of 175 events in 119 patients who fulfilled the inclusion criteria. During that time, the percentage of patients who experienced CRBSI and CC was 4.2% and 4.8%, respectively. The majority of the cohort consisted of male (59.4%), Malay ethnicity (75%), and patients on a tunnelled dialysis catheter (83%). Patients who were fistula naïve and had an internal jugular catheter were more common in the CRBSI group than in the CC group. The predominant microorganisms that were isolated were Gram-positive organisms. In terms of clinical presentation and outcome, no differences were found between the CRBSI and CC groups. Patients with Gram-negative bacteraemia, high initial c-reactive protein, and catheter salvation were likely to have poor outcomes. Recurrence of CRBSI occurred in 31% of the cohort. Neither catheter salvation nor antibiotic-lock therapy were associated with the recurrence of CRBSI. On the other hand, the femoral vein catheter site was associated with risk of recurrence. The overall mortality rate was 1.1%. *Discussion*. From the analysis, it was concluded that clinical assessment and positive culture are crucial in diagnosing CRBSI with or without peripheral culture. This study provides essential information for the local setting which will enable healthcare providers to implement measures for the better management of CRBSI.

## 1. Introduction

Chronic kidney disease is a major public health issue and a significant economic burden [1]. The prevalence of end-stage renal disease (ESRD) is increasing exponentially in Malaysia [2]. The main renal replacement therapy modality in Malaysia is haemodialysis (HD), and the majority of ESRD patients receive dialysis at stand-alone dialysis centres. The tunnelled dialysis catheter (TDC) is an important method of vascular access in patients with chronic HD, especially in a substantial proportion of the currently dialysed population who no longer have the possibility of receiving treatment via native vascular access. The use of HD catheters in Malaysia increased from 6.5% in 2007 to 13.6% in 2016 with almost an equal number of patients receiving the TDC and the nontunnelled dialysis catheter (NTDC) [2]. Haemodialysis patients are at risk of dying from cardiovascular-related complications and sepsis. Death-related sepsis has generally increased in medical practice over the years. In 2016, 28% of all deaths among HD patients was due to sepsis [2]. Also, the incidence of bacteraemia among HD patients is greater especially among those with indwelling catheters as compared to those with an arteriovenous fistula (AVF) or with a graft. Catheter-related bloodstream infection (CRBSI), exit-site infections, tunnel infections, and catheter colonization (CCs) are common complications related to central venous catheter use in HD patients. Catheter-related bloodstream infection alone has an incidence of 1.1 to 5.5 episodes per 1000 catheter days and is associated with increased morbidity, hospitalisation, and death. Among ESRD patients, those who have a TDC have a 15-fold increased risk of CRBSI and all-causes mortality of 12% to 25% as compared to those patients with an AVF [3].

The approach taken to manage CRBSI mainly depends on the type of catheter and the severity of the infection. According to the Malaysian National Antibiotic Guideline published in 2019, empirical systemic antibiotics should be administered based on the local antibiogram [4]. Therefore, following the antibiogram study conducted by Halim et al. in 2016, the empirical antibiotics that are used for HD CRBSI in the setting for this study, Universiti Kebangsaan Malaysia Medical Centre (UKMMC), are IV ceftazidime and IV vancomycin [5]. Furthermore, the decision to remove catheter should depend on the severity of the infection and the organism involved. In circumstances where catheter salvage is attempted, a systemic antibiotic and an antibiotic lock should be administered [6].

Timely and accurate diagnosis of CRBSI is crucial to reduce complications. The Infectious Disease Society of America (IDSA) established a CRBSI guideline in 2009, which is largely followed worldwide. In this guideline, the mainstay in diagnosing CRBSI is positive blood cultures from the peripheral veins and catheter hub that must all meet the quantitative or differential time to positivity (DTP) criteria [7]. However, obtaining a culture from a peripheral vein is a challenge in HD patients due to exhausted vascular access. Furthermore, many experts in the field prefer to preserve peripheral veins for future fistula creation. Hence, this rigid IDSA guidance for the diagnosis of CRBSI among HD patients is controversial because it is not validated for these patients. In a study by Quittnatt et al., a combination of venous catheter hubs and HD circuits were reported to be the most sensitive and accurate way to diagnose CRBSI as compared to peripheral venipunctures [8]. There is very limited data evaluating probable CRBSI patients with positive cultures from catheter hubs but yielding negative cultures from peripheral veins.

Given the conundrum and discrepancies between the standard and the real-world practice of defining CRBSI, our intention in this study was to evaluate the prevalence of CRBSI and CC events among of patients receiving HD via catheter in our centre, UKMMC, based on the IDSA definition and to understand the clinical characteristics and outcomes of those patients. We also sought to evaluate the factors that affected the poor outcome and the recurrence of CRBSI. We hypothesised that there would be no significant differences in the clinical characteristics and outcomes for CRBSI and CC events in UKMMC and that both may perhaps represent the true numbers of CRBSI.

## 2. Methodology

### 2.1. Study Population

We conducted a single centre, retrospective, observational study at UKMMC. The study protocol was approved by the Medical Research Ethics Committee of UKM (FF-2019-447) with a waiver for informed consent because the data that were collected from the database were secondary data. All ESRD patients aged 18 and above, who were dialysed via the catheter method and who had symptoms of suspected CRBSI (such as fever, chills, and intradialytic fever/hypotension that could not be explained by another diagnosis) were screened for inclusion in the study. Patients who were confirmed to have CRBSI and CC with positive blood culture results between 1 January 2016 and 31 December 2018 were recruited to the study. Patients with missing and incomplete data, and those with confirmed infections from other sources were excluded from the study.

### 2.2. Definition of CRBSI

For this study, we defined suspected cases of CRBSI as patients with an indwelling dialysis catheter in situ, who presented with signs and symptoms of fever (>38°C) or chills with no other obvious source of infection, where blood cultures via catheter hub and percutaneous peripheral vein were taken simultaneously and empirical antibiotics for CRBSI were administered.

In this study, CRBSI is defined as in IDSA (2009) [9], where similar organisms have been isolated from blood cultures obtained from both a peripheral vein and a catheter lumen, provided that microbial growth from the peripheral vein cultures are detected more than 2 h later in the catheter lumen using the DTP method. A CC was defined as showing significant growth (≥1 microorganism) in a quantitative (≥10^2^ CFU per catheter segment) or a semiquantitative (≥15 CFU per catheter segment) culture of the catheter hub [7]. Catheter salvation was defined as a catheter that was not removed within 2 weeks of initial diagnosis of CRBSI.

### 2.3. Data Collection

Haemodialysis patients with suspected CRBSI were identified from the pharmacy's patient database. Patients who fulfilled the criteria for CRBSI and CC were selected, and their notes were reviewed via accessing either the original physical hardcopy or the electronic medical records. Relevant patient details were collected and documented in a standardised data collection sheet. These details included demographic information such as age, gender, duration of indwelling catheter placement, type of catheter, duration of dialysis, reason for dialysis, previous medical history, previous history of CRBSI and CC, clinical manifestation, types of organism, types of antibiotics, and complications such as septic shock, metastatic infection, and mortality.

### 2.4. Statistical Analysis

All the collected data was entered into the Statistical Package for the Social Sciences version 25 software for further data cleaning and statistical analysis. Continuous data were expressed as mean ± standard deviation (for normally distributed data) or as median and interquartile range (for nonparametric data) and categorical data were presented as frequency and percentage. Categorical variables were compared using the chi-square test, whereas continuous variables were compared using the independent *t*-test (for normally distributed data) or Mann–Whitney *U* test (for nonnormally distributed data). We also used a multiple logistic regression to look for the risk factors and confounders associated with the outcomes.

## 3. Results

**Figure 1:**
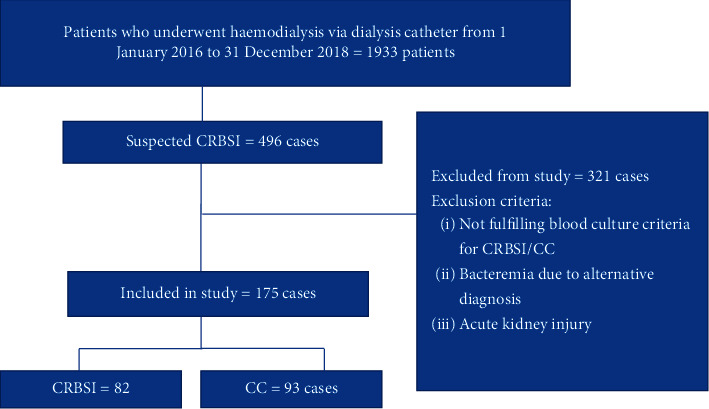
Study flow diagram.

A total of 1933 patients with TDC and NTDC underwent HD in UKMMC from 1 January 2016 to 31 December 2018 (Figure 1). A total of 496 suspected CRBSI cases were screened. However, 321 (64%) of the patients were excluded (for reasons, see Figure 1). Thus, 175 (36%) of the screened patients fulfilled the study criteria and were therefore included in the final sample.

### 3.1. Patient Characteristics

The basic demographic, clinical, and laboratory characteristics of the patients are provided in Tables 1 and 2, where they are stratified into two groups: CRBSI or CC.

The majority of patients in both the CRBSI and CC groups were male and Malay. The most common cause of ESRD in both groups was diabetes mellitus (DM) followed by hypertension.

Most of the CC and CRBSI patients were on TDC as compared to NTDC. The CC patients were mainly on a femoral vein catheter (FVC) while the majority of the CRBSI patients were on an internal jugular vein catheter (IJC). Also, there were more fistula-naïve patients in the CRBSI as compared to the CC group.

However, the analysis of patient characteristics suggested that there were no differences between the groups in terms of their clinical presentations such as fever, intradialytic hypotension, chills, and rigours or in catheter pain or pus discharge. We also found that there was no difference in their haemodynamic status upon presentation.

### 3.2. Outcomes

The outcomes of CRBSI and CC are preented in Table 3. On the whole, patients with CRBSI and CC had no statistical significant difference in their outcomes. Complications such as septic shock occurred in 18% and 12% of patients in the CRBSI and CC groups, respectively. By using a binary multivariate regression, it was statistically proven that those who had their catheter salvaged (*p*=0.026) and patients who grew a Gram-negative organism (*p*=0.028) were more likely to have poor outcomes (shock, metastatic infection, mortality, and intensive care unit (ICU) admission), as shown in Table 4. These results were obtained after adjusting the model fit to the variables of age, gender, and comorbidities. In the patients who had poor outcomes, the regression analysis showed that the initial c-reactive protein level was a factor that influenced the outcomes (*p*=0.026).

Recurrence of CRBSI occurred in 31% of the study population. Patients with preexisting DM did not have a high recurrence rate of CRBSI. Patients with a FVC were more likely to have a recurrence of CRBSI as compared to those with an IJC (Table 5). A further subanalysis on patients with FVC revealed that those who had their catheter salvaged were more likely to have a recurrence of CRBSI (OR = 2, 95% [CI = 1.006–5.115], *p*=0.048) after other variables (comorbidities: DM, hypertension, and age) were controlled. Polymicrobial organisms were also found to be statistically significant in predicting the recurrence of CRBSI (OR = 2.9 [95% CI = 1.025–8.303] *p*=0.045).

Among the study population, 72 (41%) of the catheters were salvaged and out of these, 28 (39%) had a catheter-related infection recurrence. Among patients with a salvaged catheter, nine received an antibiotic lock as an adjunctive treatment alongside a systemic antibiotic. The remaining patients received only systemic antibiotics as treatment. Among those who received the adjunctive antibiotic-lock therapy, four (44%) had a recurrence of infection during the study period, while among those who received systemic antibiotics exclusively, 24 (38%) had recurrent infection. We found no statistical significant difference in the occurrence of CRBSI in both groups (*p*=0.728).

Mortality occurred in two out of 175 cases (1.1%) in the study sample, of which both cases had Gram-negative bacteraemia. All of the cases had a femoral TDC at the time of diagnosis.

### 3.3. Microorganisms Isolated for CRBSI and CC

A total of 186 isolates were cultured from 175 patients, as described in Table 6. The majority of the blood cultures (62%) yielded Gram-positive organisms. The most common Gram-positive organisms in CRBSI group were methicillin-resistant *staphylococcal aureus* (MSSA) (26%), followed by methicillin-resistant *coagulase-negative staphylococci* (MRCONS) (25%). On the other hand, *coagulase-negative staphylococci* (CONS) (29%), *Bacillus* (22%), and *Diphtheroid* (10%) were Gram-positive organisms that were commonly found in the CC group. With regard to Gram-negative organisms, *Pseudomonas* (25.8%) was the most common organism cultured in the CRBSI group, while *Enterobacter* sp. (20%) was the most common in the CC group.

## 4. Discussion

In this retrospective analysis of 3 years of UKMMC data on ESRD patients on HD via indwelling catheter (TDC or NTDC), we observed that 496 cases were empirically treated for suspected CRBSI. However, the majority of the cases (64%) did not fulfil the IDSA blood cultures definition for CRBSI or CC and have an alternative diagnosis. Thus, only 36% were found to fulfil the above definition for CRBSI or CC. This suggests that attending clinicians have low thresholds for empirically treating CRBSI when the patients have clinical signs and symptoms of infection.

Among the 175 patients included in this study, 82 (47%) had CRBSI and 93 (53%) had CC based on the IDSA blood culture definition. These numbers are almost equivalent for the two conditions even though they have distinct definitions in the IDSA guideline. In view of the similarity in the initial clinical presentations, such as fever, chills, and rigours and intradialytic hypotension, there was a tendency to treat them empirically with intravenous systemic antibiotics while awaiting the blood culture results. This action was perhaps taken to potentially avoid dealing with a worsening case of sepsis or bacteraemia.

We also observed that during the period from 1 January 2016 to 31 December 2018, the percentage prevalence of CRBSI and CC among our cohort was 4.2% and 4.8%, respectively. The prevalence for CRBSI differs from that reported in another local study by Azila et al. in 2016 and from that reported in a study conducted in Canada in 2014 [9, 10]. The difference in the prevalence may be due to variation in the study designs and different definitions use of CRBSI and CC. Previous studies have shown that the CRBSI rates using the IDSA criteria are usually about 18% lower than the CRBSI rates using the CDC definition [11, 12].

In regard to the demographic data, those for age and gender correspond with the latest report by the Malaysia Dialysis and Transplant Register published in 2017, which showed that there is an increasing trend of dialysis among patients aged ≥65 years old [2].

Our study was designed to investigate both CRBSI and CC. Thus, all of the patients in our study who had an indwelling catheter were predominantly given a TDC (82.7%). A smaller number of patients on NTDC implies physicians' aggressive measure to reduce the HD patients' risk of CRBSI. The TDC is an important vascular access option in those patients who have exhausted vascular access and are not suitable for peritoneal dialysis [13].

The clinical characteristics of the CRBSI and CC groups were rather similar except for catheter type and history of having a previous fistula. We found that patients with a TDC and a history of AVF were more common in the CC group. These findings are consistent with those of two other observational studies [14, 15]. The greater proportion of these patients in the CC group can be explained by the use of a subcutaneous cuff in the TDC which impedes bacterial migration from the exit site to the venous entry site [16]. Moreover, we found that patients with a history of fistulae had a longer duration of HD (>12 months). Therefore, we postulated that they were likely to have exhausted their peripheral vessels, which resulted in a difficult venesection during the peripheral blood culture at the time of presentation, which then led to negative culture results. One possible explanation for the negative results is, perhaps, an inadequate amount of blood being drawn from peripheral veins for the blood culture. In their study, Quitnatt et al. reported that less than one-third of CRBSI events meet the diagnostic criteria of CRBSI using the IDSA DTP criteria. They also found that blood cultures in the HD circuit and venous hubs were far superior as compared to any combinations of peripheral vein culture with catheter hub or HD circuit cultures [8]. Thus, it is possible that the CC cohort may represent the true picture of CRBSI prevalence. This supposition may also provide a justification for the CC patients being treated with systemic antibiotics similar to the patients in the CRBSI group. This finding is extremely important for our practice, especially when dealing with ESRD patients who have already exhausted their peripheral vessels, and who are therefore unable to provide a sample for a peripheral culture. Hence, this knowledge might reduce the burden of cost incurred in undertaking peripheral cultures and reduce the failure rate in obtaining a peripheral vein culture by inexperienced phlebotomists.

In our study, we also found that 50% of the patients were on a FVC and 42% were on an IJC. The argument that femoral catheter placement is strongly associated with a higher risk of catheter infection remains controversial. The avoidance of a femoral catheter placement has been recommended in many HD guidelines [6]. In our study, catheter sites were found to be associated with a recurrence of CRBSI. This is consistent with previous studies that have suggested femoral sites of catheter insertion should be considered a high-risk factor for bacteraemia as compared to subclavian and internal jugular sites. This increased risk can be attributed to bacterial colonization within the perineal skin [17, 18]. However, in a Cathedia randomised study that involved 750 patients with IJCs and FVCs, it was reported that there is no statistically significant difference in catheter colonization or in the rate of CRBSI between both groups [19].

### 4.1. Microorganism Spectrum

With regard to the microorganisms involved in CRBSI and CC, our study findings are consistent with the literature on the growth of Gram-positive microorganisms [14, 17]. The most common isolates that were identified in our study were CONS followed by MSSA. A multicentre Canadian study showed that the majority of isolates obtained from catheter infections in TDC are Gram-positive bacteria [20]. Since both CONS and MSSA are part of the normal flora found on human skin, we can conclude that patients are more likely to be susceptible to staphylococcal infections, with vascular access being a main port of entry. The higher prevalence of Gram-positive cocci found in our study differs from the results reported by a study done in UKMMC in 2014 by Abd Halim et al., where they reported Gram-negative bacteria as the predominant microorganism [5].

### 4.2. Outcomes in CRBSI

Our analysis indicated that Gram-negative bacteraemia and salvaged catheters were significant predictors of having poor CRBSI outcomes (septic shock, mortality, metastatic infection, and ICU admission). Patients with Gram-negative organisms and those who had their catheter salvaged were three times more likely to suffer from poor outcomes. This is consistent with the findings from a study conducted in a critical care setting by Abe et al., where a significantly higher incidence of septic shock among Gram-negative as compared to Gram-positive bacteraemia patients was reported [21].

Our analysis also revealed that two-thirds of the patients who had their catheter salvaged showed no recurrence during the study period. This result is consistent with a study conducted by Damien et al., where bacteraemia in two-thirds of the cases with catheter salvage were reported to have been successfully treated [22]. This finding has significant clinical value and may be applicable in our setting, especially with regard to making decisions about whether or not to salvage infected catheters that are commonly seen in dialysis patients with exhausted vascular access, in those unsuitable for peritoneal dialysis and in those who are dialysing using the “lifesaving” tunnelled catheter.

In some circumstances where catheter removal is not feasible, catheter salvation is attempted and systemic antibiotics are administered for 2 weeks (with or without antibiotic-lock). In our study sample, there was no significant difference in the recurrence of catheter infection in the patients who had adjunctive antibiotic-lock therapy as compared to those who received only systemic antibiotics. This finding differs from that of a study conducted by Aslam et al., which reported that the antibiotic lock with systemic antibiotics is superior to systemic antibiotics alone especially in terms of recovery and recurrence [23]. The contrasting findings may perhaps be due to the smaller sample size in our study, especially in respect of the administration of the antibiotic lock.

## 5. Limitations

This was a retrospective study that was conducted in a single centre, which limits its generalisability to other settings. We were also unable to calculate the catheter days due to missing data for the catheter insertion date variable. This was due to the fact that many of the patients had their catheter insertion at other hospitals before being admitted to UKMMC for CRBSI or CC. We may also have underestimated the true rate of CRBSI for the following reasons: (1) the blood culture was occasionally obtained after starting empirical antibiotics and (2) the volume of blood drawn for cultures was sometimes minimal, hence lowering the yield of positive blood cultures.

## 6. Recommendations

Further studies with larger sample sizes are needed to enable an update of the antibiogram at UKMMC. A randomised control study with a larger sample size could be done for CRBSI treatment especially with antibiotic-lock therapy as an adjunct treatment to systemic antibiotics. This would help to determine whether there are any statistically and clinically significant differences in the outcomes of patients treated with systemic antibiotics exclusively and those treated with adjunctive antibiotic-lock therapy.

## 7. Conclusion

In conclusion, this study provides evidence that the definition of CRBSI in HD patients may differ from those in existing guidelines. Clinical assessment and a positive culture with or without availability of a peripheral culture should prompt the clinician to treat accordingly. Also, early recognition of risk factors that may lead to complications of CRBSI is important for the clinician to anticipate and reduce morbidity and mortality.

## Figures and Tables

**Table 1:** Demographic characteristics of CRBSI and CC groups.

Characteristics	CRBSI (*n* = 82)	CC (*n* = 93)
Age (median, IQR)	66 (59–72)	64 (55–73)

Gender (*n*, %)		
Male	54 (66%)	50 (54%)
Female	28 (34%)	43 (46%)

Race (*n*, %)		
Malay	64 (78%)	68 (73%)
Chinese	14 (17%)	20 (22%)
Indian	4 (5%)	5 (5%)
History of AVF (*n*, %)	47 (57%)	69 (74%)

Catheter type (*n*, %)		
TDC	63 (77%)	82 (88%)
NTDC	19 (23%)	11 (12%)

Catheter placement site (*n*, %)		
Internal jugular vein	41 (50%)	34 (37%)
Femoral vein	36 (44%)	52 (56%)
Other (subclavian vein, external jugular vein)	5 (6%)	7 (7%)

Cause of ESRD (*n*, %)		
Diabetes mellitus	63 (77%)	64 (69%)
Hypertension	12 (15%)	15 (16%)
Other	7 (9%)	14 (15%)

Comorbidities (*n*, %)		
Diabetes mellitus	64 (78%)	61 (66%)
Hypertension	72 (88%)	75 (81%)
Ischaemic heart disease	21 (26%)	24 (26%)
Dyslipidaemia	22 (27%)	21 (23%)
Peripheral vascular disease	20 (24%)	18 (19%)

Past history of dialysis catheter infection (*n*, %)	28 (34%)	44 (47%)

Duration of dialysis (*n*, %)		
<3 months	11 (13%)	7 (8%)
>3 months	71 (87%)	86 (92%)

AVF: arteriovenous fistula; TDC: tunnelled dialysis catheter; NTDC: nontunnelled dialysis catheter; ESRD: end-stage renal disease; IQR: interquartile range.

**Table 2:** Clinical characteristics and blood parameters of CRBSI and CC groups.

Clinical manifestations and blood parameters at presentation	CRBSI (*n* = 82)	CC (*n* = 93)
Fever (*n*, %)	79 (96%)	88 (95%)
Intradialytic hypotension (*n*, %)	13 (16%)	9 (10%)
Chills and rigours (*n*, %)	69 (84%)	70 (75%)
Pain at catheter site (*n*, %)	5 (6%)	3 (3%)
Pus discharge (*n*, %)	6 (7%)	8 (9%)
SBP (mmHg) (mean, SD)	141 ± 32	145 ± 29
DBP (mmHg) (mean, SD)	7 ± 17	72 ± 16
MAP (mean, SD)	94 ± 20	96 ± 19

SBP: systolic blood pressure; DBP: diastolic blood pressure; MAP: mean arterial pressure; SD: standard deviation; IQR: interquartile range. Significance value: *p* < 0.05.

**Table 3:** Outcomes for CRBSI and CC groups.

Outcomes	CRBSI (*n* = 82)	CC (*n* = 93)
ICU admission	7 (8%)	5 (5%)
Mortality	1 (1%)	1 (1%)
Catheter salvage	29 (35%)	43 (46%)
Metastatic infection	2 (2%)	0 (0%)
Recurrent CRBSI	23 (28%)	31 (33%)
Septic shock	16 (18%)	11 (12%)

ICU: intensive care unit.

**Table 4:** Factors associated with poor outcome (shock, death, metastatic infection, and ICU admission).

Factors	Poor outcome (*n* = 28)	No poor outcome (*n* = 147)	*p* value
Age (median, IQR)	63 (57–72)	66 (55–73)	0.885

Gender (*n*, %)			
Male	15 (54%)	89 (61%)	0.491
Female	13 (46%)	58 (39%)	

History of previous catheter infection	15 (54%)	57 (39%)	0.145

Comorbidities			
Ischaemic heart disease	6 (21%)	39 (26%)	0.571
Hypertension	23 (82%)	124 (84%)	0.770
Diabetes mellitus	19 (67%)	106 (72%)	0.648

Type of catheter			
TDC	24 (86%)	121 (82%)	0.790
NTDC	4 (14%)	26 (18%)	

Catheter site			
Femoral and others	19 (68%)	81 (55%)	0.211
Internal jugular	9 (32%)	66 (45%)	

Catheter salvage	17 (61%)	55 (37%)	0.026^*∗*^

Type of microorganism			
Gram positive	11 (39%)	93 (63%)	0.028^*∗*^
Gram negative	14 (50%)	46 (31%)	
Mixed growth	3 (11%)	8 (5%)	

Initial blood parameters			
Haemoglobin (g/dl) (mean, SD)	9.9 ± 1.7	9.7 ± 2	0.566
Platelets (x10^9^/L) (mean, SD)	197 ± 83	222 ± 83	0.150
White cell count (median, IQR)	11.4 (9.3–17.6)	10.5 (7.6–14)	0.06
Albumin (g/L) (mean, SD)	28 ± 5	29 ± 5	0.262
C-reactive protein (mg/L) (median, IQR)	9.3 (5.7–16.7)	5.5 (2.9–9.7)	0.026^*∗*^

^*∗*^Adjusted to age, comorbidities (diabetes mellitus, hypertension, and ischaemic heart disease). TDC: tunnelled dialysis catheter; NTDC: nontunnelled dialysis catheter; IQR: interquartile range; SD: standard deviation.

**Table 5:** Factors associated with recurrent of CRBSI after an event.

	Recurrent CRBSI (*n* = 54)	No recurrent CRBSI (*n* = 121)	*p* value
Comorbidities			
Ischaemic heart disease	15 (28%)	30 (25%)	0.677
Hypertension	42 (78%)	105 (87%)	0.134
Diabetes mellitus	32 (59%)	93 (77%)	0.036^*∗*^

Type of catheter			
TDC	48 (89%)	97 (80%)	0.157
NTDC	6 (11%)	24 (20%)	

Catheter site			
Femoral and others	39 (72%)	61 (50%)	0.049^*∗*^
Internal jugular	15 (28%)	60 (50%)	

Catheter salvage	28 (52%)	44 (36%)	0.150

Type of microorganism			
Gram positive	27 (50%)	77 (64%)	0.103
Gram negative	21 (39%)	39 (32%)	
Mixed growth	6 (11%)	5 (4%)	

Polymicrobial (≥2 organisms)	9 (17%)	8 (6.6%)	0.045^*∗*^

^*∗*^Adjusted to age, comorbidities (diabetes mellitus, hypertension, and ischaemic heart disease). TDC: tunnelled dialysis catheter; NTDC: nontunnelled dialysis catheter.

**Table 6:** Spectrum of cultured organisms in CRBSI and CC groups.

Organism	CRBSI (no. of isolates = 87)	CC (no. of isolates = 99)
Gram-positive organism (*n*, %)	57 (66%)	59 (60%)
MSSA	15 (17.2%)	5 (5.1%)
MRCONS	14 (16.1%)	5 (5.1%)
CONS	4 (4.6%)	17 (17.2%)
*Bacillus*	6 (6.9%)	13 (13.1%)
MRSA	6 (6.9%)	3 (3%)
*Diphtheroid*	4 (4.6%)	6 (6.1%)
*Enterococcus*	6 (6.9%)	4 (4%)
*Streptococcus*	2 (2.3%)	2 (2%)
Others	0	4 (4%)

Gram-negative organism (*n*, %)	30 (34%)	40 (40%)
*Pseudomonas*	8 (9, 2%)	5 (5.1%)
*Acinetobacter*	3 (3.4%)	6 (6.1%)
*Klebsiella*	3 (3.4%)	2 (2%)
*Enterobacter*	6 (6.9%)	8 (8.1%)
*E. coli*	3 (3.4%)	0
*Stenotrophomonas*	4 (4.6%)	8 (8.1%)
Others^*∗*^	3 (3.4%)	10 (10.1%)

^*∗*^Others = C*andida parapsilosis, Roseomonas gilardii, Micrococcus, Rhodococcus, Citrobacter koseri, Chryseobacterium, Serratia, Pasteurella, Burkholderia* sp.*, Corynebacterium*. MSSA: methicillin-resistant *staphylococcal aureus*; MSSA: methicillin-sensitive *staphylococcal aureus*; MRCONS: methicillin-resistant *coagulase-negative staphylococci*; CONS: *coagulase-negative staphylococci*.

## Data Availability

The data used to support the findings of this study are available from the corresponding author upon request.
